# Anticipation and response: pandemic influenza in Malawi, 2009

**DOI:** 10.1080/16549716.2017.1341225

**Published:** 2017-07-28

**Authors:** Evanson Z. Sambala, Lenore Manderson

**Affiliations:** ^a^ School of Public Health, Faculty of Health Sciences, University of the Witwatersrand, Johannesburg, South Africa

**Keywords:** Sub-Saharan Africa, health service infrastructure, influenza surveillance, H1N1 responses, pandemic planning and response

## Abstract

**Background**: In 2006, Malawi developed a national influenza plan to mitigate, prevent and manage the burden of infection should an outbreak occur. In 2009, it translated its contingency plan to respond to the unfolding influenza pandemic. However, little is known of how Malawi translated its national influenza plan into response actions, or the success of these responses.

**Objective**: To investigate how Malawi translated its preparedness plan and so broaden our understanding of the outcomes of the responses.

**Methods**: We draw on data from 22 in-depth interviews with government policymakers and people working at a policy level in various non-governmental organisations, conducted to assess the level of preparedness and the challenges of translating this.

**Results**: Through a number of public health initiatives, authorities developed communication strategies, strengthened influenza surveillance activities and updated overall goals in pandemic training and education. However, without influenza drills, exercises and simulations to test the plan, activating the pandemic plan, including coordinating and deploying generic infection control measures, was problematic. Responses during the pandemic were at times ‘weak and clumsy’ and failed to mirror the activities and processes highlighted in the preparedness plan.

**Conclusions**: Participants stressed that in order to achieve a coordinated and successful response to mitigate and prevent the further transmission of pandemic influenza, good preparation was critical. The key elements which they identified as relevant for a rapid response included effective communications, robust evidence-based decision-making, strong and reliable surveillance systems and flexible public health responses. To effectively articulate a viable trajectory of pandemic responses, the potential value of simulation exercises could be given more consideration as a mean of sustaining good levels of preparedness and responses against future pandemics. These all demand a well-structured planning for and response to pandemic influenza strategy developed by a functioning scientific and policy advisory committee.

## Background

The dominance of highly pathogenic avian influenza (HPAI) epidemics in poultry, such as influenza A (H5N1) virus in the late 1990s, sharpened a perception of the threat of an influenza pandemic in human populations [1,2]. In response, the World Health Organization (WHO) issued warnings of the potential of serious disease through animal-to-human transmission, and expressed concern that once a pandemic had started, it would be too late to undertake many of the activities required to minimise its impact [3]. These threats of an imminent pandemic constituting a ‘Public Health Emergency of International Concern’ led to the publication of guidelines to assist its member states to prepare for a new pandemic in 1999 [3].

Following the outbreak of severe acute respiratory syndrome (SARS) in 2003, firstly in southern China and various Southeast Asian countries, then worldwide, the WHO [4] requested that all countries develop pandemic preparedness plans based on newly revised guidelines. These guidelines were revised again 2009, 2011 and 2013, incorporating knowledge and lessons learnt from the outbreaks of SARS, HPAI (H5N1) and the 2009 H1N1 outbreaks [5,6]; for original guidelines see [3]. Most countries in Africa responded by developing plans aimed at reducing the transmission and threat intensity of pandemic influenza across rather than within national borders [7].

Malawi developed its first influenza implementation plans in March 2006, and revised it in 2009, in accordance with the International Health Regulations [4], with a focus on human influenza subtypes [8]. Prior to this, there was an incident in Ntchisi, central region of the country in December 2005, where thousands of sick migratory birds (fork-tailed drongos) dropped dead from the sky, precipitating fear of an avian flu outbreak in the country. The large wetlands around the Lake of Malawi provide a high risk of HPAI, creating an ideal breeding ground for the avian virus in seasonal migratory birds. As a result of these threats, an avian preparedness team was established, led by the Ministry of Agriculture (MoA) and supported by the Ministry of Health (MoH).

In 2006, the MoH took over responsibility for managing, updating and evaluating all aspects of the pandemic plan [9,10]. This new plan to prevent influenza in humans included changes in the roles, coordination and leadership of an influenza taskforce and committee as the focus changed from avian to human influenza. The national plan, confirmed in 2006, was based on existing operational structures at the national level, including health service infrastructure, health committees, surveillance networks and coordination, and command structures. In the implementation plan, for example, district hospitals were designated to manage influenza cases, rather than all cases being referred to Influenza Assessment Centres (IACs).

In January and February 2009, as a global pandemic appeared imminent with the active circulation of the H1N1 influenza virus in parts of America, authorities warned countries to be alert. The first recognised case of 2009 H1N1 influenza was officially declared in mid-April 2009 in Mexico, by which time the influenza virus had been actively spreading for some months. The 2009 pandemic influenza was mild but fatal in children, pregnant women and people with underlying chronic illness. Epidemiological data on 2009 pandemic influenza in Malawi is scarce and during the 2009 pandemic influenza no death related to influenza was reported. The lack of reporting reflects underreporting and how poorly the infection was understood. Pandemic influenza is a global threat, and killed over 50 million people globally in 1918.

When WHO declared the pandemic on 11 June, 2009, a national taskforce was established in Malawi comprising technical experts in infectious diseases. The taskforce met regularly to discuss and provide scientific advice on the management strategies and operational emergency responses required should a pandemic be declared in Malawi. Despite these meetings, and the existence of a formal plan, progress towards influenza preparedness across the country was slow, with the MoH facing numerous practical challenges [7]. These included the lack of an effective influenza surveillance system to send early signals of impending influenza activity in human and animal populations, and the lack of mechanisms to provide information to health services at the district level for the prevention, treatment and control of influenza. These operational challenges raised concerns about whether specific responses to planning for and response to pandemic influenza (PRPI), as characterised in the national plan, were achievable. This problem was not unique to Malawi [7].

Since the 2009 pandemic outbreak, scholars have analysed how some countries in Africa responded [11,12] and identified lessons from the global preparedness responses [13]. However, little is known of how Malawi translated its national influenza plan into response actions, or the success of these responses. To our knowledge, the study we report here is the first to investigate the planning for and responses to the 2009 pandemic influenza in a low income country, where governance, infrastructure and health systems might all compromise health outcomes. In this article, we focus on the challenges and operational problems Malawi encountered. We draw on interviews conducted with policymakers involved in PRPI to broaden our understanding of country preparedness and the outcomes of the responses. We conclude by identifying areas where preparedness and future responses might be improved.

## Methods

### Study design and setting

Malawi is a land-locked country across the East African Rift Valley, bordered by Mozambique in the south and east, Zambia to the west and Tanzania to the north and northeast. The country has an estimated population of 17.2 million (2015), and while characterised as a developing market economy, it is consistently among the poorest nations in the world, with a GDP of around USD 900 per capita per annum. Health expenditure per capita per annum is around USD 29 (2014), accounting for 8.4% of total percentage of GDP; this amount is insufficient to meet the costs of essential health services provided by the MoH. The health system is therefore dependent on donor aid and there is no public social health insurance system. As a least-resourced country, Malawi provides a significant case study to assess how severely limited budgets constrain pandemic preparations and the response actions that arise from such efforts.

Malawi was identified from among the 46 African countries that attended the first Regional Conference on Pandemic Influenza A (H1N1) in 2009, in which the first author took part. Malawi was purposively selected based on its economic status, geographical location, influenza surveillance system and the availability of a national pandemic preparedness plan. These considerations included a number of points of comparison with other countries, as indicated above, including low health expenditure and health system limitations. Malawi was among the first countries in Africa to develop pandemic plans, but their implementation was not known until now. Malawi was also feasible and practical to collect data because it is politically stable and safe, and English is widely spoken, so allowing interviews to be conducted and data analysed without translation assistance. The comparison with other countries and triangulation of information derived from this setting provides the basis for validity.

In choosing Malawi for the study, we also considered the fact that any severe pandemic influenza outbreak would have adverse effects and human suffering (including economic disruption) on vulnerable and ‘at risk’ populations. Resource poor countries like Malawi are at increased risk because of limited access to prevention and treatment interventions, and large subpopulations are particularly vulnerable because of underlying health conditions [14]. Malawi is likely to be heavily affected by influenza because of its large immunocompromised population due to HIV. In addition, inadequate public health infrastructure, overcrowding, poor sanitation and living conditions heighten any risk of a pandemic outbreak and severity of impact [15].

The research on which we draw was a qualitative and descriptive study, conducted to gain knowledge of policymakers’ experiences and insights from their accounts of pandemic influenza planning and responses. This information will assist in providing a better understanding of how resource-poor countries can plan for and respond to any pandemic, including, for instance, Ebola, Chikungunya or Zika as well as future influenza pandemics.

### Participants and sampling

Policymakers who were involved in the PRPI, including representatives from both government (macro policymakers) and non-governmental agencies (micro policy actors) were interviewed. All were in relevant positions of power or authority prior to the pandemic, and so influenced or determined policies. Government policymaker interviewees were drawn from infrastructure ministries and other bodies that formed the executive arm of the government, including politicians and others with political appointments, members of boards, officers within government ministries, researchers and managers directly serving the government. The micro level representation in the policy framework on PRPI included executive directors, managers and scientists from local and international non-governmental organisations (NGOs). All were identified from a pool of heterogeneous actors in governments, civil society and NGOs, who were able to comment and voice their opinions on how the country had planned and responded to the pandemic.

A total of 29 policymakers were identified directly and through snowball sampling [16] for interviews, although five were subsequently excluded because of their lack of direct involvement in PRPI activities, and two policymakers declined to participate. In the end, 22 participants were interviewed. All participants were contacted first with an official letter, information sheet and consent form, sent to them either by email or fax.

### Interview process, instrumentation and policy documents

In-depth interviews were conducted between January 2012 and January 2013, with this data complemented by a review of Malawi’s pandemic preparedness plans, including the plan that was current at the time of the 2009 pandemic and subsequent revised versions. A question guide for a relatively unstructured interview was adopted, enabling the interviewer to cover essential points, while modifying the order of questions depending on the context and flow of conversation. This method provided flexibility for respondents to critique, comment, explain and share their experiences, opinions and attitudes as they wished. The interview guide included the following questions: (1) How would you describe the influenza preparedness plan before 2009? (2) How did you translate the pandemic plan into response actions during the 2009 pandemic outbreak? (3) What were the main operational problems, challenges and lessons learnt from the 2009 pandemic? and (4) How did you resolve problems during the 2009 pandemic? Before each interview, the researcher introduced himself and explained the purpose of the interview. Interview duration varied but ranged from 48 to 145 minutes (mean 70). Extended interviews afforded us in-depth data rather than breadth data that would cover all themes. This enabled us to build a convincing analytical narrative [17]. We were interested in individual perceptions and narratives of the processes, policies and programs, not in generalisability, and in this context, we were not concerned with generalising among participants.

The interview locations varied according to agreed arrangements. These locations were generally safe and quiet so as to avoid disturbances. The interviews were mainly conducted in the interviewee’s office, cafes and hotel lobbies. The majority of the interviews were held at the interviewee’s convenience, generally during their lunch hour or after work. All interviewees completed a profile form with personal data and the role they played in PRPI. Written consent was provided by all participants, and all interviews were audio-recorded. The study was approved by the Medical School Research Ethics Committee of the University of Nottingham, UK (where the first author was a student) and the MoH in Malawi, to ensure informed consent and confidentiality.

### Data analysis

After each interview, the first author listened and re-listened to audio recordings to gain familiarity with the data and enable iteration, and entered notes into a data analysis logbook. Note taking was repeated after full transcription, during the coding of the data, and again at the time of writing. All transcribed interviews were exported to NVivo 8 to facilitate coding and thematic organisation. We used the six thematic areas of preparedness and responses set out in the WHO checklist [18] as a framework of analysis to identify respondents’ views on the levels of preparedness, responses, strengths and weakness. These included planning and coordination, surveillance, communication, public health interventions, patient management and maintaining essential services. With these thematic areas in mind, coding was adopted, with the text examined closely, line by line, to identify both pre-defined and new themes. Generating codes involved three stages. The first was open coding, where interesting features of the data were identified, labelled and defined. Initial codes matching data extracts were collated by labelling and assigning a selection of unique identifiers of text within each data item. The second phase proceeded by reviewing and refining themes in which the connections between concepts (such as planning and public health infrastructure) were explored to help build categories and interrelationships. Here, we considered whether predefined themes and sub-themes formed a coherent pattern and if not, whether this was problematic. Themes that did not fit particular data extracts were redefined or discarded. The third phase involved searching selective coding, where predefined themes were further defined and refined. Throughout the process, codes identified in the data, predefined themes and categories were subjected to an iterative process, involving constant testing of data, confirming or negating the concepts, until the patterns in the data were clearly understood. This provided structure to the extracted data and interpretation in the final analysis.

## Results

As noted, 22 policymakers were interviewed. The broad categories of analysis that policymakers primarily referred to were challenges and operational problems encountered when responding to a pandemic influenza. As we discuss below, six themes from the analysis on PRPI were identified: governance and decision-making; coordination and advice; politics, science and policy; key infrastructure for PRPI; information, communication and education; and prevention, mitigation and containment. Responses arising from preparations and actions undertaken during the pandemic period included activating the pandemic plan and initiating influenza surveillance measures. They also included the deployment of generic infection control advice, and coordinating and communicating different response actions in order to maintain the routines and functions of civil society.

As noted above, the MoH led the coordination and execution of the pandemic plan at the national level from early 2009 and activated it in April 2009, when the first cases of pandemic influenza were reported in Mexico and when its spread to other continents and countries became a growing global concern. An influenza working group within the MoH coordinated the development of the country’s plan and actions to address the pandemic. Response activities were supported by implementing agencies with different roles (Table 1).

**Table 1.** Implementing agencies in PRPI.

Task	Responsible (Partners)
Technical assistance	World Bank/FAO/OCHA/MoH/WHO
Advocacy	USAID/UNICEF/WHO
Funding	World Bank/FAO/OCHA/WHO
Policy development	MoH/WHO/MoA
Implementation	MoH/CHSU /WHO/MRCS
Coordination, monitoring and evaluation	MoH/CHSU/MoA/DoDMA/CHAM
Logistics	MoH/UNDP/World Bank/FAO/OCHA

CHAM: Christian Health Association of Malawi; CHSU: Community Health Sciences Unit; DoDMA: Department of Disaster Management Affairs; FAO: Food and Agriculture Organization; MRCS: Malawi Red Cross Society; MoA: Ministry of Agriculture; MoH: Ministry of Health; OCHA: Office for the Coordination of Humanitarian Affairs; UNDP: United Nations Development Programme; UNICEF; United Nations International Children’s Emergency Fund; USAID; United States Agency for International Development; WHO; World Health Organization.

The development of the Malawi country plan was guided by the WHO PRPI strategy, based on pandemic phases and the conceptual framework of public health functions including communication, surveillance, logistics, detection and response, and containment. The WHO recommended that member states consider a six tier inventory of pandemic phases when developing or updating the national plan – an inter-pandemic period (Phase 1 and 2), pandemic alert period (Phases 3, 4 and 5) and pandemic period (Phase 6) – in the context of country-specific needs, priorities and actions. Malawi made some country-specific modifications to the WHO PRPI strategy and reduced the WHO six-tier inventory of pandemic phases to three tiers: alert, serious and emergency (Table 2).

**Table 2.** The three-tier structure operating in Malawi.

Three-Tier Response Levels	Public Health Actions
**Alert**	
(a) Highly pathogenic avian influenza (HPAI) detected in poultry population outside the country. (b) Avian influenza human cases detected outside the country.	– To obtain timely and accurate information from other places with a view to prevent introduction of the disease into the country and to detect local cases as early as possible.
**Serious**	
(a) Current strain of the virus arrives in the country via migratory birds and quickly infects local domestic bird populations in specified geographic foci. (b) Due to the low rate of bird-to-human transmission of the virus, relatively few human cases are detected.	– To contain the disease as soon as possible, identify foci of infection, prevent local transmission and exportation of disease to other places.
**Emergency Response Level**	
(a) viral strain capable of rapid and effective human-to-human transmission. (b) Influenza pandemic declared by WHO.	– To contain the disease as soon as possible, identify foci of infection, prevent large outbreak from occurring, interrupt and stop chain of local transmission and prevent exportation of disease to other places. – To slow down progression of the epidemic and minimise loss of human lives in order to buy time for the production of an effective vaccine against the novel pandemic influenza strain.

Source: Government of Malawi [9].

These three-tier response levels were based on different risks and predicted a course that might be taken in the event of an avian influenza pandemic. The three tiers, according to the research participants, eased communication with the general public, but the typology was considered irrelevant by some participants because its main focus was on avian influenza to the neglect of other types of influenza. As a result, participants were not clear how they might have used this framework in the context of the influenza outbreak spreading in humans. Table 3 provides a summary of the themes and recommendations.

**Table 3.** Themes and recommendations.

Theme	Recommendations
Governance and decision-making	Need for sustainable influenza funding and development of command structures that must not heavily rely on external funding.
	Need for efficient and timely decision-making from policymakers in the Ministry of Health in order to offer guidance on public health policy on influenza.
Coordination and advise	Need for plans at the local level that engage local people, families and medical personnel to ensure local services are running smoothly during the pandemic period.
	Need for private and public partnership to continue providing essential services such as water, energy and safe transport.
	Need for external research cooperation and reinforce ongoing cooperation.
	Responsibilities and actions needs to be defined phase by phase.
Politics, science and policy	Need to vaccinate timely (seasonal and pandemic).
	Need for influenza research focusing on the national and local context in order to manage challenges and problems anticipated during the influenza outbreak.
	Border control such as screening need to be improved.
	Need for political intervention to improve pharmaceutical logistics in acquiring vaccines and other drugs.
	Need for effective hospital control policies.
Key infrastructure for PRPI	Need for strengthening health services operations and making sure non pharmaceutical (hygiene and sanitation) and pharmaceutical products (vaccines and antibiotics) for mitigating influenza are available on time.
Information, education and communication	Need for an effective programme to change public attitudes and perceptions about influenza.
	Need to strengthen communication by electronic means, phone, and meetings.
	Need for communicating real time and hypothetical surveillance data.
	Need for communicating the nature, spread, peak and decline of influenza (seasonal and pandemic).
Prevention, mitigation and containment	Need for Influenza Like Illnesses (ILI) case investigation by interviewing patient cases and carrying out surveys for possible sources and make public aware that ILI is reportable.
	Need for surveillance working groups and need for reporting absenteeism in work place and schools.
	Upgrade laboratory networks and diagnostic capacity including active sentinel surveillance through the Integrated Disease Surveillance and Response (IDSR) and other operational structures like FluNet.
	Need for influenza web reporting systems.
	Need for rapid test technology in rural areas.

### Governance and decision-making

Participants drew on diverse vocabularies to describe their influenza preparedness plan and their own understandings of preparedness. One participant (P17) described the plan as a ‘milestone’, indicating significant achievements in national pandemic influenza planning activities. Another explained: ‘We came up with the flu plan pretty quickly and updated overall goals in pandemic flu training, communication, monitoring and surveillance’ (P3). The majority of participants, however, described the preparedness plan as ‘clumsy’, ‘incomplete’, ‘weak’, ‘unreliable’ and ‘frustrating’, with one participant commenting that the preparedness plan was ‘short-lived’. One person emphasised that the plan had been abandoned ‘when the pandemic was at its peak in November 2009, due to lack of funding’ (P10).

Some participants commented on the absence of risk scenarios of disease severity in the plan, and lack of clarity of goals in preparedness and response to the needs of particularly vulnerable groups, cities and towns. Participants noted the lack of clarity around services outside the health portfolio, for instance, on how to continue to keep schools open in the context of a pandemic, or how water companies might continue to provide clean water. Others, however, referred to the value of the basic scenarios, for example, how best to facilitate food security to avoid food shortages, and how to increase service output in health facilities so that general health care was not compromised. Participants emphasised that the effectiveness of preparedness was not just a matter of having a plan, but of being able to respond and find legitimate solutions to the pandemic. As one ministerial participant, who openly expressed disappointment, remarked: ‘What is the point of having a plan that does not work?’ (P2).

Malawi moved quickly on the declaration of the pandemic. A special advisory committee on pandemic influenza was established to oversee the implementation of the pandemic plan, and country delegates joined other African countries at the first African Regional Conference on Pandemic Influenza A (H1N1) in Johannesburg, held in June 2009, to discuss the implementation of the plans. Malawi also participated in the deployment of H1N1 vaccines funded by the WHO and other donors, an action not part of the initial preparedness plan but incorporated in response to donor recommendations. As part of the response, Malawi established influenza surveillance at ports of entry, with routine checks for suspected cases, and suspected samples were sent to Kenya for diagnostic confirmation. Hence, as one participant remarked, ‘despite the lack of labs, we managed to set up surveillance networks within the country and this paid off by picking up four confirmed cases’ (P21). The MoH was also seen to be successful in sensitising the public on the risks of the pandemic and the means of prevention, through its use of advertisements on national radio and the distribution of written educational materials to the general public.

### Coordination and advice

Despite weak infrastructure in the health system and limited management capacity, the command structures of the PRPI, including the advisory committee, were in place to lead coordination and leadership for the early detection and rapid containment of influenza. However, respondents reported that the advisory committee was unable to provide streamlined operational and expert advice; it remained focused predominantly on probable threats of avian influenza rather than the antigenic shifts of both avian (H5) and human influenza subtypes. Preparedness prior to 2009 H1N1 had failed to account for other influenza viruses subtypes, such as H1, H2, H3, H7 or H9, although most policymakers believed these subtypes posed pandemic risks too. In addition, many respondents viewed the advisory committee as non-representative, and considered it neither ‘transparent’ [P2] nor ‘inclusive’ [P17]; they felt that the government could have better engaged its partners in establishing a working advisory committee to meet the different needs of the people represented by different organisations. Participants also expressed concern that the experts appointed to advise on influenza preparedness were not specialists in infectious disease control and not qualified to perform their duties. As one participant argued, ‘I think the [advisory] committee was hastily instituted without bringing on board experts who know more about the topic [influenza, preparedness and response]… not a good beginning for an efficient and effective planning system’ [P13].

In addition, respondents from some of the participating organisations, outside of the MoH, felt undermined and excluded in the planning process due to financial conflicts and fights over budgets within the government departments. Such conflict led to departments other than Health distancing themselves from the activities of PRPI.

### Politics, science and policy

In order to gain perspective on how PRPI was shaped, respondents were asked about the factors that influenced the PRPI plan and implementation. Three core narratives emerged: the influence of politics, the science of pandemic planning, and the strategic policy process related to pandemic preparedness. Respondents argued that politicians wanted to tackle the influenza pandemic at a political level, without the best available knowledge. Those involved in preparedness planning argued the importance of high-quality knowledge in policy processes, while scientists emphasised the importance of evidence-based operational tasks to elicit successful outcomes. Respondents felt that there was a ‘blame game’, with key actors accusing each others of inadequate actions. Policymakers within the MoH felt that politicians dominated preparedness at all levels of government; those respondents who were politicians meanwhile claimed that department level policymakers were passive. Scientists meanwhile claimed they were not involved in the preparation of the plans, and this limited the validity of the plans: ‘Pandemic solutions lie within scientific knowledge, norms and research’ (P19).

Both scientist and policy making respondents pointed to corruption and maintained that political approaches were geared to self-interest as much as a lack of knowledge on pandemic control. This limited the ability of policy actors to make decisions objectively. Some respondents argued that politicians directly influenced the activities of PRPI, citing the vaccination programme in particular as politically motivated. For example, they claimed that politicians wished to create an impression that the vaccination uptake was high, and so forced members of target groups to receive the vaccine against their will. One respondent argued that the decision to force people to be vaccinated was made to avoid throwing away unused vaccines; others maintained that this coercion, involving the police, stigmatised the very population meant to be protected, while ‘milking money’ out of the WHO which was funding the vaccination programme.

### Key infrastructure for PRPI

Most respondents conceded that the country had taken reasonable steps to achieve the goals of the plan, but were disappointed in the response actions and identified areas for improvement. One respondent reflected, ‘We did *something*, but of course not very well, because most of the activities we had embarked on were affected by the lack of critical infrastructure’ (P8). Another respondent, a lay person who was involved in implementation, argued that preparedness required the use of the available infrastructure, such as existing surveillance systems and laboratories. However, in Malawi, key infrastructure was severely limited, hence the use of diagnostic laboratories in Kenya. Establishing new infrastructure such as laboratories during emergency situations is practically impossible, given that this would take several years. For most respondents, the only way to achieve responses that mirrored preparedness plans was to improve health services generally, but essential services to facilitate rapid diagnostic, care and monitoring of disease spread all needed strengthening. A few respondents identified existing infrastructures, such as the Health Management Information System (HMIS), an information technology system that could have been deployed and adapted to provide timely information on influenza outbreaks. Respondents across the interviews were aware that having a functional information technology system would ensure the easy flow of information about the pandemic outbreaks, specifically to inform public health policy about, for example, whether additional staff were needed. Several suggested linking this with the integrated disease surveillance response (IDSR) system for management of health information, including planning for and management of health services.

### Information, education and communication

Respondents noted that there were communication problems in disseminating information about the pandemic. For example, national authorities failed to warn districts, regions and local areas about the possible spread of the disease after the official outbreak announcement in Mexico, and one ministerial respondent said that scant attention was paid to educating the public on the need to contain infection. Respondents felt that the government and collaborating organisations could have done more to explain the cause, pattern of transmission, and impacts of the disease. An international agency representative commented that: ‘Officials never sufficiently stressed the nature or duration of the pandemic in their preparedness plans, its spread, its peak and decline, nor did they sufficiently inform the public on these issues’ (P11). Another ministerial policymaker maintained that due to communication problems, the local implementation team was unclear when to implement the plan, when to make vaccines and non-pharmaceutical interventions (NPIs) available, and when to evaluate the interventions.

The policymakers who criticised the handling of pandemic plan and the communication process proposed new ways to improve information, education and communication (IEC). For example, they suggested the use of multi-media communication, including setting up telephone hotlines and social networking sites such as Facebook and Twitter. A respondent from a bilateral aid agency suggested email updates as part of response strategy. These are all options available in the country, although they are also expensive for consumers and not available to all people.

### Prevention, mitigation and containment

Respondents believed that authorities failed to set up influenza specific services to minimise the impact of the pandemic on hospitals and other social institutions. Although wider community interventions such as closing schools were not considered appropriate given the scope of the pandemic, respondents insisted that infection control measures such as encouraging hygiene and sanitation should have been implemented. Most respondents were quick to mention that influenza infrastructure systems were crucial in maintaining operations of surveillance, mitigating and responding to the disease. In reflecting on the implications of this for continued country capacity to respond to pandemics, respondents argued that there was a need for a national influenza centre and/or regional IACs to carry out monitoring and assessment: ‘To respond effectively, we need an influenza virology laboratory. This will assist with virus isolation and subtyping of the virus’ (P1). Many respondents emphasised the value of procuring bio-security and bio-safety equipment, particularly influenza diagnostic equipment and reagents, to test influenza samples in local laboratories.

Respondents emphasised that influenza prevention, mitigation and management could have been strengthened had specialists been able to develop treatment protocol and tighten hospital communication and surveillance network systems, including monitoring and evaluation of response actions, and adequate training: ‘Training of specialised personnel, such as laboratory technicians, clinicians and epidemiologists, was needed for effective public health responses’ (P12).

Participants suggested that in future, monitoring seasonal influenza activities might be a predictive indicator to aid estimates of additional capacities needed to detect increase in pandemic activity. Respondents suggested the maintenance of health service infrastructure, including planning for ‘loss of workforce’ (P5), ‘absenteeism’ (P19) and additional health staff in working hours when regular staff failed to turn up for work.

## Discussion

From 2006, in anticipation of an outbreak of pandemic influenza, Malawi drafted a preparedness plan that would assist in reducing its threat and intensity. Although there was substantial preparedness planning and progress in its cycle of development, many planning tasks and responses remained unmet. The 2009 HIN1 pandemic influenza arrived sooner than was expected, and the plan was activated in April 2009 with inadequate time for influenza drills, exercises and simulations to test the plan. Surveillance at the time, however, included only screening for possible influenza cases at country borders. Activities were incomplete prior to and during the pandemic, and were characterised by poor reporting, lack of surveillance systems and infrastructure. Adequate reporting would have required building the capacity of the virology reference laboratory to conduct appropriate tests and collect appropriate and timely data to characterise the epidemic by person, place and time. This can be done through influenza web-based reporting systems and where possible, the use of rapid test technology in rural areas to capture real time surveillance data.

There were inevitable tensions between global policy and its local operationalisation. The adoption of decisions without consideration of Malawi-specific situations, needs, priorities and actions created confusion and communication difficulties. Insufficient epidemiological evidence at the national and local level raised questions about the science behind PRPI, echoing Schuchat’s and colleagues’ argument about decision-making with imperfect science, and the challenges of generalising patterns of transmission or risk factors in seasonal and pandemic influenza [19]. In this study, we found a weak scientific advisory committee unable to bridge the gap between the epidemiology of the disease and operational responses. This reflects in part the role of simulation exercises which could identify priorities and actions, and provide opportunities to test the level of preparedness. Simulation exercises may be integral to improving decision-making, procedures and skills by identifying gaps and weaknesses in the PRPI.

Participants identified lack of coherence between scientific knowledge of pandemic influenza and operational advice during the response period, and suggested that how the pandemic unfolded pointed to conflicts in the development of the pandemic plan and its implementation. The plan was not mirrored in the responses undertaken; nor were responses locally informed. New WHO recommendations on pandemic influenza were released halfway through the unfolding pandemic crisis, and these required an overhaul and changes to the pandemic preparedness plan. Malawi could not adequately consider the newly proposed WHO recommendations on pandemic phases to respond effectively to the pandemic outbreak.

The issues relating to scientific advice have broad ramification, touching on several aspects of the relationship between planning for and coordinating pandemic influenza responses. Lack of epidemiological data may impact on authorities’ level of action. In this study, participants maintained that the ministerial authorities directly involved in the planning were passive when epidemiological data, resources and critical infrastructure fell short. According to Parsons, social agents (i.e. authorities able to take action) and social structures (i.e. surveillance and health systems) are both important determining factors for action [20]. Holm also reiterates that decision-making occurs not in a vacuum, but by social agents at large, by the general social environment, and by organisational features such as those of health care institutions [21].

As we have argued, scarce resources limited the capacity of the government to strengthen the health system to ensure that responsibilities and infrastructure were adequately supported. A number of important weaknesses were identified in the robustness of influenza surveillance systems intended to send early signals for health service response, including the lack of efficient and timely decision-making among policymakers to guide public health policy on influenza. The WHO recommends reinforcement of routine surveillance capacity both from an epidemiological and virological standpoint to monitor influenza. This requires collection of sickness reports combined with laboratory and clinical investigations as valuable indicators of the occurrence of influenza. It is essential to monitor for influenza-like illnesses (ILI), including severe acute respiratory infections (SARI) during influenza season and non-season periods. Unless these ILI and SARI are characterised and monitored, policymakers will not be well informed on the burden of respiratory diseases in order to prioritise resources and plan public health interventions to mitigate influenza infections. Institutions within existing systems, such as hospitals and laboratories, need to document and assess influenza data. This can be undertaken using surveillance techniques such as reporting ILI or SARI, and absenteeism in work place and schools.

Any pandemic influenza outbreak, regardless of severity, can lead to intense pressure on health services when a high number of sick people need attention. At the same time, pandemic influenza is a serious threat, with a large population at risk of infection in a relatively short period of time. Even mild to moderate illness in the population can significantly disrupt social life and economic activities, with one study suggesting that pandemic influenza could reduce GDP by 0.5–4.3% [22]. A WHO study conducted in Thailand, Uganda and South Africa, for example, suggested that the 2009–2010 pandemic influenza contributed to a GDP loss of up to 0.05% of these countries [23]. In many respects, where resources are limited, pandemic influenza can be responded to in the same way as seasonal influenza and other respiratory infections such as TB. However, if PRPI is to be sustainable and sensitive to context, there is a need for internal funding and the development of command structures that do not heavily rely on external funding. Business continuity planning is required by both health and non-health sectors to complement and consolidate national plans, and to ensure continuation of the vital day-to-day functions of the society. For example, a business plan in the health sector may need to consider alternative power sources, if routine supplies affect operations due to constant surges, blackouts, and/or chronic shortages of fuel.

In interviews, participants explained that the planned risk scenarios and general preparedness were based on H5N1/avian influenza, raising questions of the uncertainty of interventions largely targeting human influenza. Proper assessment of the pandemic risk is needed in any context to alert decision-makers to issues related to timely response and guidance on public health policy on influenza. Due to the lack of country funds for the PRPI, policymakers fully endorsed and adopted the WHO universal guidelines on pandemic influenza, without taking into account the local context. We argue that it is not possible to simply apply a graded series of responses to emerging pandemic viruses; this requires the Pandemic Severity Index (PSI) to be calibrated to the case fatality ratio to determine actions ranging from limited to stringent measures [24].

Although Malawi’s capacity to respond was limited, there was strong commitment from government departments, international agencies and local NGOs to achieve optimal outcomes. For example, early in the pandemic, authorities decided to raise awareness among the travelling public, and instituted contact tracing activities and field investigations. However, these activities continued only for a short period and were then suspended due to lack of funding. Such decisions highlight the need for consistent public health actions to aid early detection and control. Difficulties in governance reflect inadequate preparedness for a number of planning activities, including vaccination and behaviour communication. In Malawi much needs to be done for future planning and responses to pandemics, with the need for substantial improvements in preparedness in key areas such as surveillance, robust science-based decision-making and a flexible public health response system to respond to crises.

Local planning should address alternatives where resources are scarce or unavailable. We acknowledge the financial implications of PRPI activities; however, we believe that PRPI is less about resources and more about actions at the local level. For example, communication problems encountered in 2009 could have been addressed with simple available communication tools such as telephone. According to Vaughan and Tinker, the success of planning for and responding to pandemic influenza rests clearly on three inter-related themes – information, education and communication [25]. Good responses require good planning in terms of making necessary information about the pandemic, and its severity, available to the public. Inappropriate communications and insufficient planning can greatly compromise influenza risk reduction.

Apart from practical and operational problems associated with communication, in this study we found that lack of communication was associated with poor leadership among policymakers. This supports Moore’s and Dausey’s findings about the relationship of strong leadership to effective reporting and implementation of PRPI activities [26]. Strong leadership demands evidence-based decision-making for appropriate and timely action in the case of an outbreak.

Linked to this, we identified the need to strengthen the health system because it is within this system that treatment and prevention are delivered. While financial and human resources are also needed to operate surveillance activities that support regular diagnoses of influenza and monitoring seasonal-like influenza through sentinel surveillance and laboratory tests, setting up and managing a good surveillance system for influenza in Malawi would not be a small task. Thus we suggest a number of cheap alternative types of surveillance could be used to survey and monitor influenza. For example, Outpatient Illness Surveillance was not emphasised by authorities in clinics or hospitals, and no information was stored or collected on flu patients visiting these institutions prior to the pandemic. No efforts were made to engage with alternative providers, which might have been critical had hospitals and clinics been overwhelmed with an unusually high number of patients. Preparedness plans need to take account of how the health sector can engage with different publics, traditional healers or volunteers from non-health sectors to reduce the demand and burden of patient care in case of surge turnout in crisis situations.

Apart from practical and operational problems such as lack of finances to assist in coordinating PRPI activities and operational strategies, responses were also influenced by the reported lack of capacity of authorities for PRPI. Tasked authorities were meant to ensure that advance preparations were timely and consistent with the impacts of a pandemic; this role requires highly skilled and responsible leadership. Consistent with findings by Ortu and colleagues [7] of pandemic plans elsewhere in Africa, there were serious gaps and mistakes in government efforts, priorities and service objectives impacting PRPI, and little political will from politicians. For example, the planning process laid out a one-off budget system as a cheap option for response activities, but, as argued in an editorial in *Nature*, this is an unsustainable way to address pandemic threats in the 21st century [27]. The 57th WMA General Assembly held in South Africa emphasised that the importance of political will to fund public health preparedness is paramount to dealing with pandemic threats [28].

Having effective and efficient laboratories in the country depends on how well these areas are funded. It is critical that countries have local influenza systems in place, in addition to the IDSR, to improve surveillance activity, situation monitoring, assessment and reporting. Viral surveillance is important to assist and facilitate prompt detection of influenza A viruses and other highly infectious viruses, in order to accelerate the implementation of effective public health responses [29]. Preventing and controlling influenza outbreaks in domestic animal populations are also important.

Where influenza cases are detected in the human population, implementation efforts should be directed towards NPIs such as quarantine, closing schools and hygiene promotion, although it is important to be cautious of overestimating the benefits of these strategies [30]. NPIs are only helpful in delaying infection and reducing the burden of the disease; they do not necessarily halt the disease once it is circulating. There are also concerns about how much they intrude on personal liberties. During the 2009 H1N1 pandemic in Malawi, people were forcibly vaccinated [31], despite evidence that voluntary vaccination, quarantine and isolation is possible and effective. A study in Canada found these measures were effective in Toronto during the SARS outbreak when over 27,000 affected persons were asked by public health officers to accept quarantine measures and the populace cooperated [32]. Gostin and Berkman argue that effective communication is critical for gaining public trust and participation in community containment measures [33], as would be needed for volunteer vaccination. According to Peny, authoritarian approaches not only raise ethical concerns but damage the public trust in police and health services [34].

## Limitations

This study had methodological limits related to the conduct of interviews and data analysis. The study was undertaken mainly with policymakers involved in PRPI, and their perception of policy making and program implementation reflects their own standpoints and roles. There is also a potential for recall bias, since the study was conducted three years after official recognition of the beginning of the pandemic and one year after it was declared over. The contribution of lay people, and more junior government employees and other actors, on these policies and programmes, was not included. While we attempted to recruit a representative sample, some policymakers directly involved in developing the plan were unavailable or failed to consent to participate.

## Conclusion and recommendations

Assumptions in the pandemic plan for Malawi were that planned interventions would address the pandemic outbreak in a straightforward manner, yet there are considerable discrepancies at the level of pandemic preparedness and actual responses. Although discrepancies are expected in any pandemic planning nationally or internationally due to the uncertainty associated with pandemics, it is important to have a consistent basis for planning, especially if it is to be applied at both local and national levels. While Malawi developed communication strategies, strengthened influenza surveillance and updated overall goals in PRPI, most response actions addressing the 2009 pandemic failed to achieve the important public health goals that the plans set out. We found that the national pandemic plan was not updated regularly and experts in influenza emergency management were rarely consulted. A number of gaps in national action were also identified, including poor coordination between national policymakers with local stakeholders, weak leadership in the influenza working committee and lack of surveillance structures such as IACs and diagnostic laboratories. There is need for coordination between the private and public sectors in order to continue providing essential services such as water, energy and safe transport. Cooperation on influenza activities would reinforce the implementation of PRPI, but this would require that responsibilities and actions are defined phase by phase. Influenza research focusing on the national and local context is important to manage challenges and problems that might be experienced during the influenza outbreak. In addition, there is need for political interventions to improve pharmaceutical logistics which in turn would improve the availability of vaccines and other drugs, enabling people to be vaccinated on time during seasonal and pandemic periods. However, there is also a need to ensure that the public is aware that influenza is a reportable disease. Most importantly, there is a need to upgrade laboratory networks and diagnostic capacity, to include active sentinel surveillance through the Integrated Disease Surveillance and Response (IDSR) and other operational structures like FluNet. In general, PRPI operations can be strengthened through effective IEC activities such as communicating real time surveillance data or communicating the nature, spread, peak and decline of influenza (both seasonally and during pandemics) to the general public. This can be done by electronic means, phones and meetings. The regular dissemination of such information may change public attitudes and perceptions about influenza.

Our findings corroborate mounting evidence that planning and response are only as good as the assumptions on which they are based. For example, planning at the national level can address issues at that level, but may overlook the need for planning to begin locally and that they always impact the local level. Local plans at the district level are needed to engage local communities and medical personnel to ensure local services are run smoothly during the pandemic period. Scott has suggested that emergency management begin at the local level because disasters are local [35]. In this study, policymakers identified finances as barriers to planning, and while we appreciate the significance of what money does, PRPI cannot be determined only by its budget. A number of financial agreements may need to be established with developmental partners in order to strengthen capacity in areas such as education and to resource laboratory surveillance. There is also a need to maximise resources to work in such a way to address the pandemic problem, and to identify leaders, partners and structures to implement pandemic influenza activities.

While the 2009 pandemic is past, there is a lot to learn from this experience. The way planning and actual responses were rolled out throughout the pre and post pandemic period demand renewed strategies to improve PRPI activities for future outbreaks. A PRPI model needs to be developed to achieve meaningful responses. Simulation exercises would be a particularly valuable tool to improve preparedness to ensure that specific tasks, functions and skills are met in real pandemic scenarios. Simulation exercises act as a policy tool that informs policymakers to take proactive and timely actions as part of response measures. A PRPI model that also takes into consideration the ‘prevention of ethical problems’ will enable policymakers to evaluate interventions that may maintain operational readiness during a pandemic. Most importantly, lessons could be drawn regarding the need to strengthen health systems for district health service planning in compliance with infection control standards and PRPI themes such as communication, monitoring and surveillance, and prevention. The best way to respond effectively to a pandemic is to prepare for the worst. Being prepared for, and responding to, influenza pandemics requires having well-trained epidemiologists, a functional public health service, reliable surveillance system, laboratories, and efficient communication channels. These can be supported by a functioning scientific and policy advisory committee to bridge the gap between the epidemiology of the disease and operational responses.

## Paper context

Little is known of how Malawi translated its national pandemic influenza plan into response actions during the 2009 pandemic influenza outbreak. We illustrate that responses failed to mirror the planned activities to prevent, mitigate and contain pandemic influenza. To achieve best responses would require a well-functioning health and surveillance system, including a scientific and policy advisory committee to recommend best practices and guide public health measures.
